# Supplementary material for: Investigation of the Reaction between a Homemade PEEK Oligomer and an Epoxy Prepolymer: Optimisation of Critical Parameters Using Physico–Chemical Methods
Source: Polymers (Basel). 2024 Mar 11;16(6):764. doi: 10.3390/polym16060764 (PMC10975252; doi:10.3390/polym16060764)
Supplement: Supplementary file 1 [file polymers-16-00764-s001.zip › polymers-2850610-supplementary.pdf]

polymers

MDPI

Supporting Information

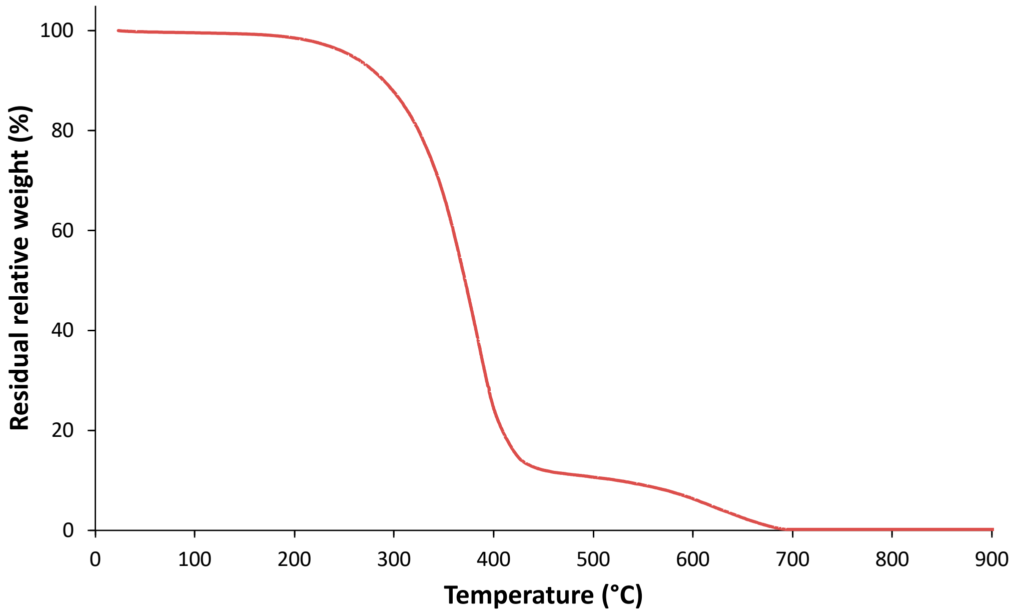

Figure S1. Thermogravimetric analysis of DER332 prepolymer under air (3°C/min).

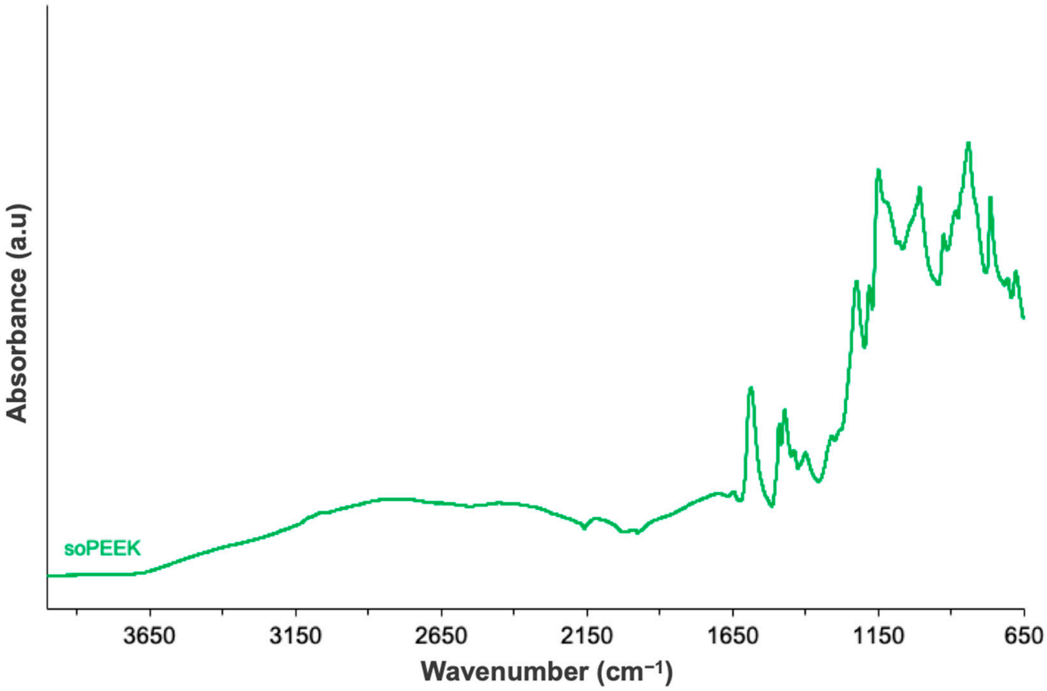

Figure S2. FTIR spectrum of soPEEK.

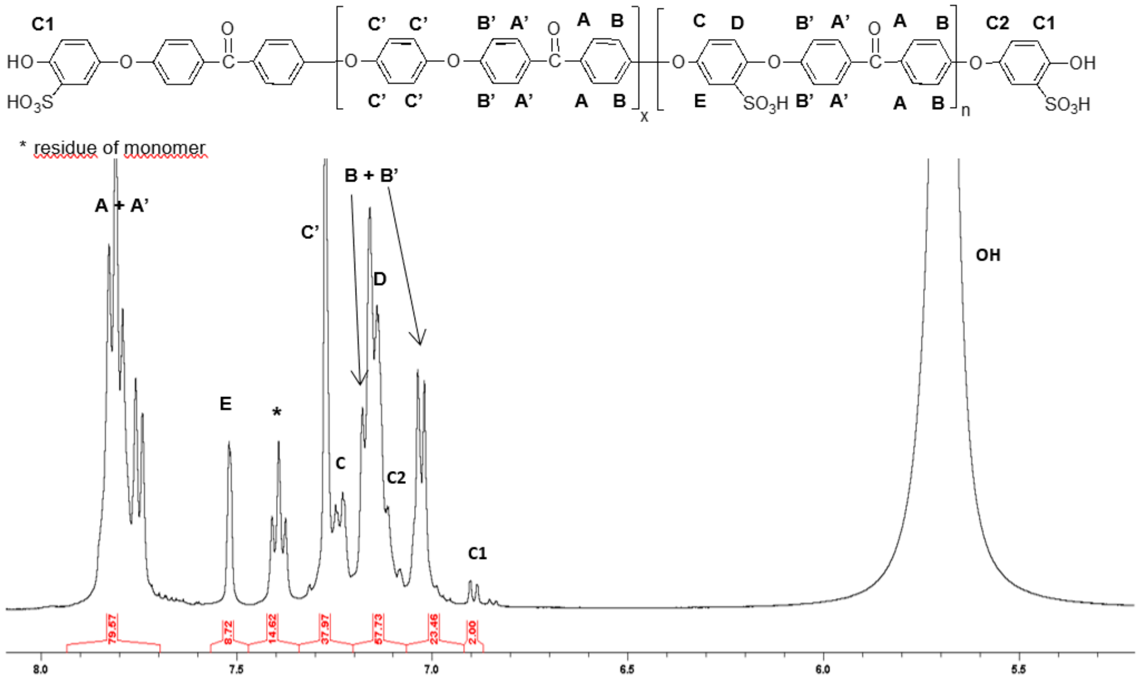

Figure S3. ^{1}H NMR spectrum of soPEEK.

The signal associated with the proton H^{E} in β-position of the $-SO_3H$ group is visible at δ = 7.52 ppm, implying that the sulfonation reaction has occured. The doublet confirms this result at δ ≈ 7.01 ppm, shifted upfield by the proximity of the $-SO_3H$ group and attributed to the protons $H^{B'}$. In addition, the signals at δ = 7.30 - 7.20 ppm, attributed to the protons H^{C} and $H^{C'}$, correspond to unsubstituted hydroquinone rings together with substituted ones in the polymer backbone. Lastly, the chain-end protons H^{C1} are visible at δ ≈ 6.90 ppm. Given the experimental conditions and especially the stoichiometry of the reactants, it is reasonable to assume that the polymer is end-capped with a hydroquinone ring on both sides. Due to their relative accessibility, it is reasonable to consider that both end rings are sulfonated. Based on this hypothesis, we could estimate the sulfonation degree (SD) of the soPEEK as well as its molecular weight and, therefore, that of the initial oPEEK. To this aim, we considered that soPEEK comprises two blocks: a sulfonated block n and a non-sulfonated block x, as represented in Scheme S1.

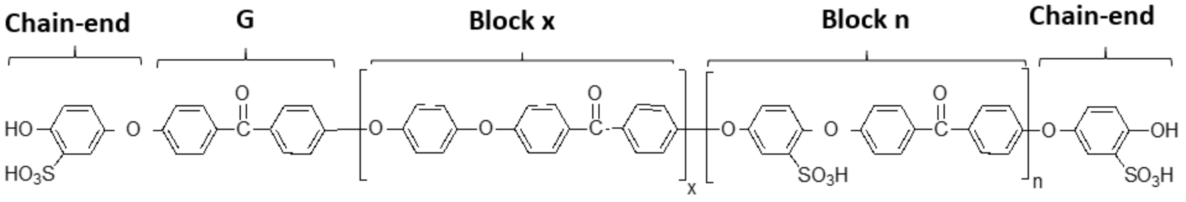

Scheme S1. Representation of soPEEK.

The polymerisation degree (PD) of block n corresponds to the number of protons H^{E} (Figure S2). According to the ^{1}H NMR spectrum, n is equal to 8.72 (Scheme S1). The PD of block x may be deduced from the integration of signals corresponding to four protons H^{A} and $H^{A'}$ in the spectral range δ = 7.70-7.90 ppm (Figure S2). However, it is necessary to consider the presence of monomer residue and the fact that H^{A} and $H^{A'}$ are also present in block x. The PD of block x is then found to be equal to 7.51. An SD value of 53% was thus found, according to Equation S1:

$$SD = \frac{PD_n}{PD_x+PD_n} \times 100 \tag{S1}$$

This value is in accordance with the SD equal to 47%, obtained from Equation S2 [1]:

$$\frac{n}{(12\text{-}2n)} = \frac{I_{H^E}}{\sum I_{H^{A,A',B,B',C,D}}} \quad (0 \leq n \leq 1) \tag{S2}$$

where n is the HE number per repeat unit (Scheme S1). Then, $M_{n,\ NMR}$ of soPEEK can be calculated from Equation S3 presented below:

$$M_{n,\ NMR}\ (soPEEK)=2 \times M\ (chain\text{-}end)+PD_x \times M\ (block\ x)+PD_n \times M\ (block\ n)+M\ (G) \tag{S3}$$

where M (chain-end) is the molecular weight of soPEEK chain-ends (i.e. 189 g/mol), PDx and PDn are the polymerisation degree of block x (7.51) and block n (8.72), respectively. M (block x) and M (block n) refer to the molecular weight of block x (288 g/mol) and block n (368 g/mol). M(G) is the molecular weight associated with the group named G (180 g/mol) (Scheme S1). According to this calculation, the average molecular weight of sulfonated oPEEK was evaluated to be close to 5900 g/mol. Then, it becomes possible to estimate the molecular weight of starting oPEEK according to equation S4:

$$M_{n,\ NMR}\ (oPEEK)=1 \times M\ (HQ)+(PD_x+PD_n+1) \times M\ (block\ x) \tag{S4}$$

where M (HQ) refers to the molecular weight of hydroquinone unit (i.e., terminated group) (110.11 g/mol). PDx, PDn, and M (block x) remain unchanged.

According to Equation S4, $M_{n,\ NMR}$ (oPEEK) = 5070 g/mol. This value is in good agreement with the targeted molecular weight based on Carother's equation, which was close to 5700 g/mol, which indicates that the expected oPEEK was finally obtained.

It is noteworthy that the assumption that there would be only one sulfonated hydroquinone end-group would yield an SD = 53%, a molecular weight of soPEEK of 2900 g/mol, and lastly, oPEEK with $M_{n,\ NMR}$ equal to 2445 g/mol, following the same calculations. These results support our first hypothesis about the complete sulfonation of both end-groups.

Reference

1. Zaidi S.M.; Mikhailenko S.; Robertson G.; Guiver M.; Kaliaguine S. Proton conducting composite membranes from polyether ether ketone and heteropolyacids for fuel cell applications *J. Membr. Sci.* **2000,** *173*, 17–34 – DOI: 10.1016/S0376-7388(00)00345-8
